# Success of an interdisciplinary educational program on nursing knowledge about epidural analgesia in a critical care setting

**DOI:** 10.1186/2197-425X-3-S1-A928

**Published:** 2015-10-01

**Authors:** ME O'Connor, T Livingstone, M Healy, A Beane

**Affiliations:** Barts Health NHS Trust, ACCU, London, United Kingdom; Barts Health NHS Trust, London, United Kingdom

## Introduction

Nursing education is a key component to the delivery of safe epidural analgesia in the post-operative critical care setting [1].

An audit of our practice revealed a significant failure rate in epidural analgesia in elective major abdominal surgery patients and a high proportion of missing documentation in cases where epidural analgesia had failed.

## Objectives

To devise and deliver an interdisciplinary education program to enhance nursing knowledge and management of epidural analgesia in critical care patients.

To improve patient safety through earlier recognition of adverse events by encouraging improved monitoring of epidural analgesia.

## Methods

We developed a teaching program for nursing staff on the care of patients receiving epidural analgesia.

The program consisted of a lecture course and interactive teaching session with a pre course questionnaire to assess existing knowledge of the benefits and complications of epidural analgesia and management of common side effects. We repeated the questionnaire after the course without the participants having prior knowledge that this would occur.

## Results

The group of 36 nurses who participated had been qualified for a median of 5 years IQR(3,10), they had worked in a critical care environment for a median of 2.5 years IQR(1, 5.25) and 25% of them had some experience of managing epidural analgesia in an environment outside of critical care.

Following the education program there was 56% increase in the number of nurses who could identify at least two advantages of epidural analgesia for patients who had undergone major abdominal surgery.

There was a 26% improvement in the number of nurses able to identify signs of high sensory level blockade, 47% improvement in the number of nurses able to name methods for improving an inadequate block and a 67% improvement in the number of nursing staff being able to identify signs of a potential epidural haematoma.

## Conclusions

Baseline nursing knowledge of clinical assessment and recognition of associated complications of epidural analgesia was limited despite a pre-existing trust-wide education program.

Interdisciplinary training is beneficial to the acquisition of short-term knowledge with regard to optimising effective pain relief and identifying common side effects.

The post course short answer exam highlights a significant increase in nursing awareness of potential serious complications of epidural analgesia.

We expect that increased nursing knowledge of the benefits of epidural analgesia and its potential problems will lead to comprehensive monitoring, superior post-operative analgesia and a reduction in adverse events.

**Figure 1:**
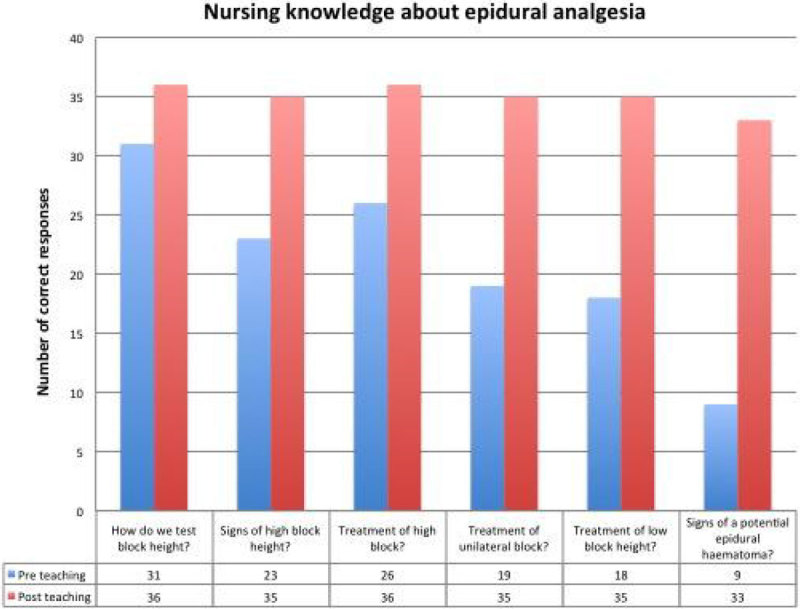
**Nursing Knowledge About Epidural Analgesia**.
